# Species-Specific Standard Redox Potential of Thiol-Disulfide Systems: A Key Parameter to Develop Agents against Oxidative Stress

**DOI:** 10.1038/srep37596

**Published:** 2016-11-21

**Authors:** Arash Mirzahosseini, Béla Noszál

**Affiliations:** 1 Department of Pharmaceutical Chemistry, Semmelweis University, Budapest, Hungary; 2 Research Group of Drugs of Abuse and Doping Agents, Hungarian Academy of Sciences, Hungary

## Abstract

Microscopic standard redox potential, a new physico-chemical parameter was introduced and determined to quantify thiol-disulfide equilibria of biological significance. The highly composite, codependent acid-base and redox equilibria of thiols could so far be converted into pH-dependent, apparent redox potentials (*E*’°) only. Since the formation of stable metal-thiolate complexes precludes the direct thiol-disulfide redox potential measurements by usual electrochemical techniques, an indirect method had to be elaborated. In this work, the species-specific, pH-independent standard redox potentials of glutathione were determined primarily by comparing it to 1-methylnicotinamide, the simplest NAD^+^ analogue. Secondarily, the species-specific standard redox potentials of the two-electron redox transitions of cysteamine, cysteine, homocysteine, penicillamine, and ovothiol were determined using their microscopic redox equilibrium constants with glutathione. The 30 different, microscopic standard redox potential values show close correlation with the respective thiolate basicities and provide sound means for the development of potent agents against oxidative stress.

Glutathione (γ-l-glutamyl-l-cysteinyl-glycine, GSH) is an important participant of the physiological defense system against reactive oxygen species (ROS), the agents of oxidative stress. In the GSH driven antioxidant actions of enzymes such as glutaredoxin, GSH is oxidized to glutathione-disulfide (GSSG). Glutathione is a key factor in the maintenance of the redox homeostasis of biological systems, due partly to its cellular concentration exceeding that of other antioxidants. The concentration of GSH ranges between 0.5 and 10 mM^1^, and is largely maintained in its reduced form by glutathione reductase^2^,^3^. Glutathione is also responsible for the regulation of thiol-disulfide balance in proteins and biogenic thiols^4^,^5^.

Another reason why glutathione works as ideal redox buffer is its optimal, pH-modulated redox potential, which is not yet thoroughly understood and quantified. Nevertheless, the ubiquitous GSH-GSSG redox system is the most widely studied one^6^,^7^. It does not necessarily require enzyme catalysis to function, and can be applied in aqueous media under a wide range of conditions. These advantages make the GSH-GSSG system a “gold standard” in thiol-disulfide biochemistry; hence, every thiol-containing antioxidant is compared to glutathione^4^. It is a key feature of any thiol-disulfide redox system, however, that only the deprotonated thiol species are active in the redox process, i.e. only the anionic thiolate can be oxidized directly^8^,^9^,^10^,^11^ (Fig. 1A). The redox potential of thiol-containing biomolecules is usually doubly pH-dependent. Primarily, the deprotonated fraction of every thiol depends on the pH of the solution, with strong dependence typically in the 6–11 pH range^12^. One clear exception to this typical acid-base behavior is ovothiol A with extremely low thiolate basicities^13^, which extends the general basicity range for thiolates to 1–10 log units in terms of protonation constants. Secondarily, the thiolate oxidizability in most biomolecules is modulated by the adjacent basic groups, since protonation of any such group exerts an electron-withdrawing effect on the thiolate. It was recently shown^14^ that in a molecule of *n* + *1* basic sites the thiolate has 2^*n*^ different, distinct oxidizabilities within a single, covalently unchanged molecular skeleton. Thus, macroscopic physico-chemical parameters cannot quantify the thiolate moiety specifically. A thorough characterization of the thiol-disulfide equilibria can be achieved by means of species-specific, so-called microscopic parameters, which are, in fact, well-established terms for acid-base systems^15^,^16^.

The direct measurement of the redox potential for thiol-disulfide systems, by usual electrochemical methods is not feasible due to formation of stable metal-thiolate complexes at electrode surfaces^17^. Thus the redox potentials of GSH and other thiols can only be determined indirectly by measurement of equilibrium constants for their reaction with redox systems of known redox potentials^7^. However, until now only the apparent redox potential of GSH was determined, largely because the highly composite species-specific acid-base properties, which are in codependent interference with the redox behavior, were not known. In this work, the species-specific standard redox potentials are introduced and applied for the most important biogenic thiol-disulfide redox couples. The systems studied are as follows: glutathione-glutathione disulfide (GSH-GSSG), cysteamine-cystamine (CysASH-CysASSCysA), cysteine-cystine (CysSH-CysSSCys), homocysteine-homocystine (hCysSH-hCysSShCys), penicillamine-penicillamine disulfide (PenSH-PenSSPen), ovothiol A-ovothiol A disulfide (OvSH-OvSSOv). The constitutional formulae of the thiols are in Fig. 1B.

The general scheme of thiol-disulfide redox equilibria (as depicted in Fig. 1C) is generally studied at the level of phenomena; the three conditional equilibrium constants are in equations (1)–(3), 2, 3 (the symbols in square brackets mean total concentration, which actually are the sum of the concentration of all microspecies of the compound in question):


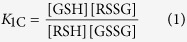



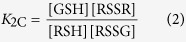



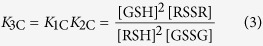


In order to get a clear insight into the redox equilibria, purified from the protonation effects, an improved evaluation method was introduced to determine the species-specific redox equilibrium constants (*k*_1_, *k*_2_, *k*_3_)^14^. The microscopic redox equilibrium constants of cysteamine, cysteine, homocysteine, penicillamine, and ovothiol A with glutathione are *sine qua non* components to determine species-specific standard redox potentials, and have been reported previously^14^,^18^. This work is the first attempt to comprehensively characterize thiol-disulfide redox equilibria in terms of the true ‘standard’ redox potentials, which are in fact pH-independent.

## Experimental Section

### Materials

1-Methylnicotinamide chloride, glutathione, and all other chemicals were purchased from Sigma-Aldrich (Saint Louis, MO, USA) and used without further purification.

### Electrochemical measurements

The redox electrode (Radeklis OP-6123, Radeklis, Budapest, surface area ~2 cm^2^) used for electrode potential measurements was washed with 50% nitric acid, then distilled water before each measurement and gently dried by touching onto tissue paper. The redox electrode was calibrated using ZoBell’s solution (2.64 g K_4_[Fe(CN)_6_].3H_2_O and 2.06 g K_3_[Fe(CN)_6_].H_2_O dissolved in 500 mL pH = 7 0.15 mol/L Na_2_HPO_4_/KH_2_PO_4_ buffer). The electrode potential measurements were carried out using a Radelkis Laboratory Digital pH/mV meter OP-211/2 (Radelkis, Budapest) at 25 ± 2 °C in an 815-PGB glove box (Plas-Labs Ic., Lansing, MI, USA) under N_2_ atmosphere to preclude oxidation by air. All potentials were referred to saturated calomel electrode as reference electrode. The pH values of the samples were determined using a Metrohm 6.0204.100 combined pH glass electrode, calibrated by aqueous NBS standard buffer solutions.

### Preparation of solutions for equilibrium constant determination

Acidic (pH = 0.85) and basic (pH = 13.15) stock solutions containing glutathione and 1-methylnicotinamide were prepared in an 815-PGB glove box (Plas-Labs Ic., Lansing, MI, USA) under N_2_ atmosphere to preclude oxidation by air. The concentrations of the reagents were optimized for quantitative NMR, ca. 15 mmol/L. A series of solutions with different pH values were prepared by mixing the acidic with the basic stock solutions. D_2_O, DSS (sodium 4,4-dimethyl-4-silapentane-1-sulfonate), glutathione reductase, and a pH indicator, which also served as a concentration standard, were added to the solutions. The samples were protected from sunlight and kept in the glove box under N_2_ atmosphere (25 ± 2 °C) for 1–2 hours, until the reactions in all the samples had reached equilibrium. To make sure that equilibrium had been achieved, NMR spectra were measured for 3 h after the reactants were combined. It was found that equilibrium had always been reached by the time the first spectrum was measured (10–20 min after mixing).

### NMR spectroscopy measurements

NMR spectra were recorded on a Varian 600 MHz spectrometer at 25.0 ± 0.1 °C. The solvent in every case was an aqueous solution with H_2_O:D_2_O, 95:5, v/v (0.15 mol/L ionic strength), using DSS as the reference compound. The sample volume was 600 μL, pH values were determined by internal indicator molecules optimized for NMR^19^,^20^. ^1^H NMR spectra were recorded with the WET solvent suppression sequence (number of transients = 64, number of points = 16384, acquisition time = 851.968 ms, relaxation delay = 15 s).

### Data analysis

**Figure 2:**
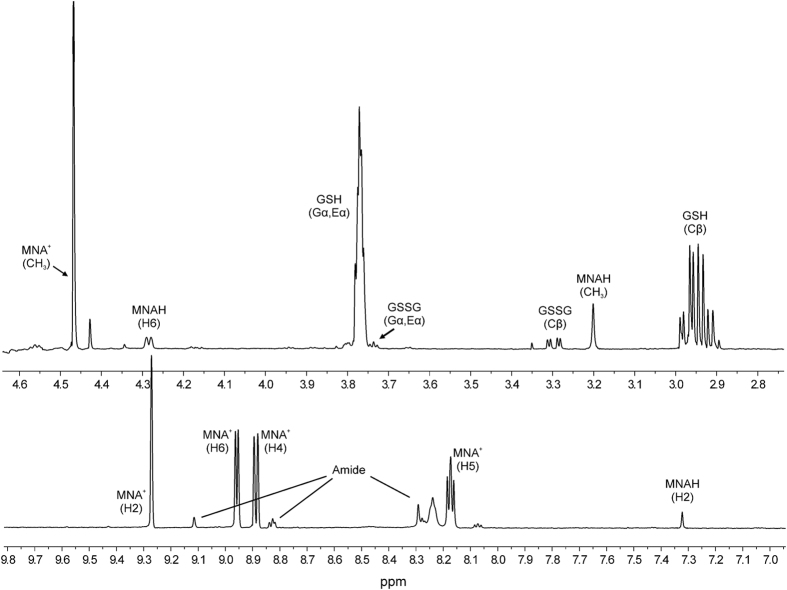
The expanded ^1^H NMR spectrum of a sample containing GSH-MNA^+^ mixture (pH = 7.04). Gα, Eα, and Cβ denote the glycinyl, glutamyl α, and cysteinyl β protons of glutathionyl residues.

For the analysis of quantitative NMR measurements (sample spectrum in Fig. 2), the peak fitting algorithm (without apodization) of the ACD/NMR Processor Academic Edition v12.01 software package (Advanced Chemistry Development, Toronto, ON, Canada) was used. For the regression analyses, the software Origin Pro 8 (OriginLab Corp., Northampton, MA, USA) was used. The standard deviations of the peak areas obtained by fitting Lorentzian peak shapes, the error of pH determination, and the standard errors of the microscopic redox equilibrium constants^17^,^18^ were used to calculate the Gaussian propagation of uncertainty to the standard redox potentials derived in the Results chapter.

## Results

**Figure 3:**
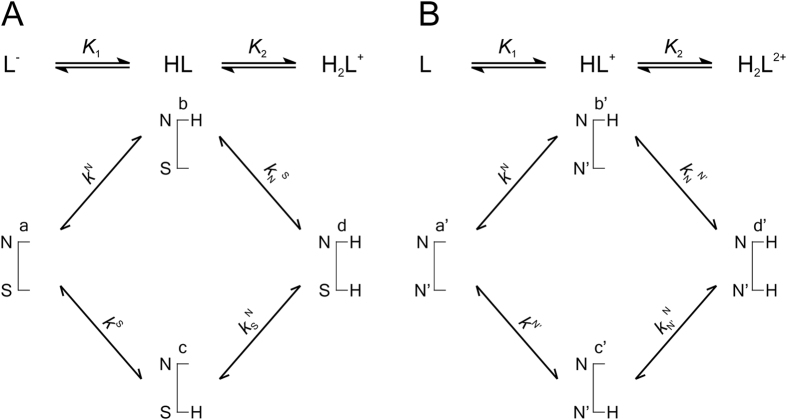
The protonation macro- and microequilibrium schemes of CysASH (**A**) and CysASSCysA (**B**) in terms of stepwise macroscopic protonation constants (*K*_1_, *K*_2_), where L^−^, HL, etc. are the successively protonating ligands (top lines). Below are the species-specific protonation schemes in terms of microspecies (a, b, c, d) and microscopic protonation constants (*k*^N^, *k*_N_^S^ …).

**Figure 4:**
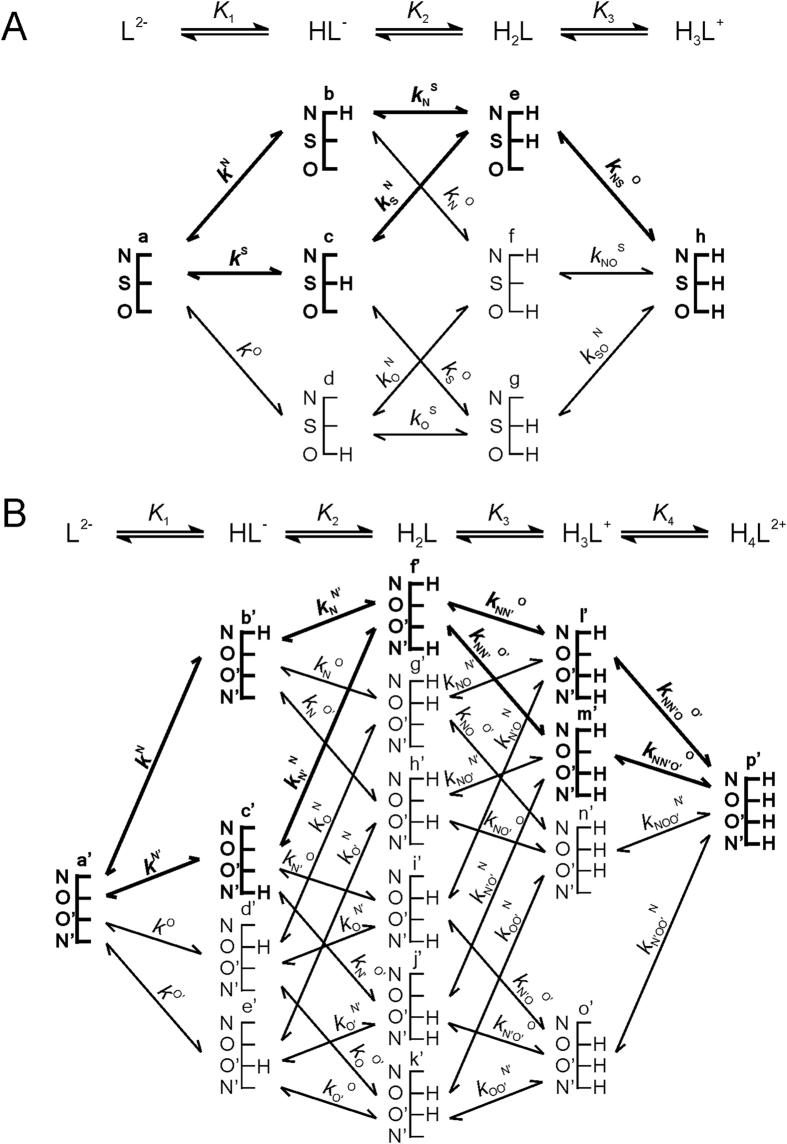
The protonation macro- and microequilibrium schemes of CysSH/hCysSH/PenSH (**A**) and CysSSCys/hCysSShCys/PenSSPen (**B**) in terms of stepwise macroscopic protonation constants (*K*_1_, *K*_2_, *K*_3_ …), where L^2−^, HL^−^, etc. are the successively protonating ligands (top lines). Below are the species-specific protonation schemes in terms of microspecies (a, b, c …) and microscopic protonation constants (*k*^N^, *k*_N_^S^ …). The components of the major pathways are in bold.

**Figure 5:**
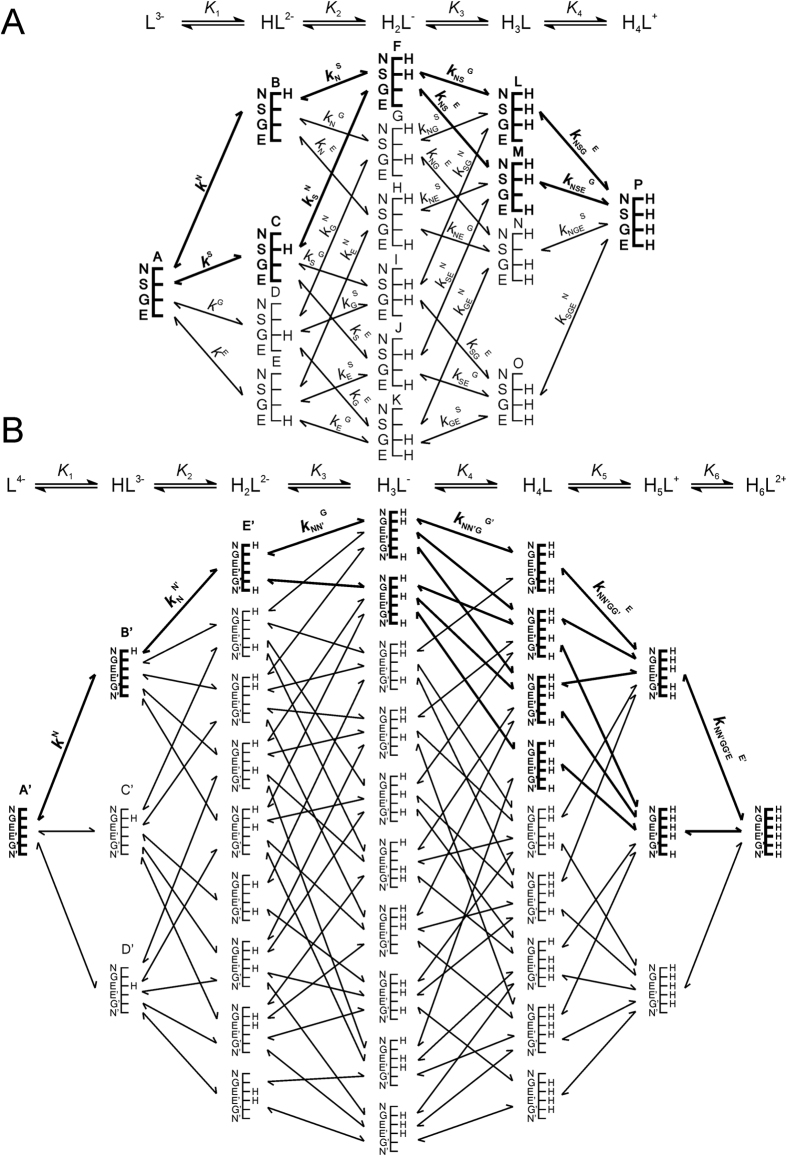
The protonation macro- and microequilibrium schemes of GSH (**A**) and GSSG (**B**) in terms of stepwise macroscopic protonation constants (*K*_1_, *K*_2_, *K*_3_ …), where L^3−^, HL^2−^, etc. are the successively protonating ligands (top lines). Below are the species-specific protonation schemes in terms of microspecies (**A, B, C** …) and microscopic protonation constants (*k*^N^, *k*_N_^S^ …). For GSSG, only non-identical microspecies and microconstants are shown, and only a few of the microconstants are depicted. The components of the major pathways are in bold. The protonation schemes of OvSH and OvSSOv are identical to the depicted schemes except Im (imidazole) and O (carboxylate) moieties are present instead of G (glycincyl carboxylate) and E (glutamyl carboxylate).

Figures 3, 4 and 5 represent the species-specific protonation scheme of the studied thiols and their respective homodisulfides. Macroequilibria (top lines) indicate the stoichiometry of the successively protonated ligand and the stepwise macroscopic protonation constants. In the microspeciation schemes, the different microspecies with their one-letter symbols (a, b, c …), and the microscopic protonation constants are depicted (*k*^N^, *k*_N_^S^ …). The superscript at *k* for any microconstant indicates the protonating group while the subscript (if any) shows the site(s) already protonated. S, N, O, G and E symbolize the thiolate, amino, carboxylate, glycinyl- and glutamyl carboxylate (the latter two pertaining to glutathion residues) sites, respectively. The acid-base microequilibria are inevitable constituents in the identification of the microspecies and the evaluation of the species-specific, pH-independent parameters. Some protonation constant examples for cysteine are shown below:


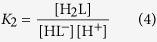



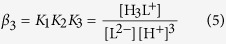



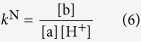


where *K*_1_, *K*_2_, *K*_3_, are successive macroconstants, *β*_3_ is one of the cumulative macroconstants, *k*^N^ is the microconstant of cysteine involved in the production of microspecies b. The concentrations of the various macrospecies comprise the sum of the concentration of those microspecies that contain the same number of protons, for example in GSH:


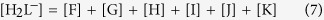


Determination of the redox potential of the GSSG/GSH system was achieved indirectly, applying a redox couple with a known standard redox potential. For this purpose the biologically ubiquitous NAD^+^/NADH (or NADP^+^/NADPH) system could be a straightforward choice. However, based on the structure and triprotic nature of NAD^+^ it was anticipated that the protonation of these moieties will influence the already pH-dependent redox potential of the system. Based on preliminary results (data not shown) the chemical shift of the pyridinium ring-bound hydrogens vary greatly with the protonation of the basic moieties, therefore the electron density around the pyridinium ring is certainly perturbed. The redox behavior of the NAD^+^/NADH system is therefore influenced by 1) the pH of the solution, since hydrogen ions participate in the redox half-reaction, 2) the 8 protonation states of NAD^+^, due to its three basic moieties, 3) the 16 protonation states of NADH, due to its four basic moieties (two pyrophosphate moieties, an adenine ring, and an added dihydropyridine ring). Therefore, the simplest nicotinamide nucleotide analogue system, 1-methylnicotinamide (MNA^+^, the oxidized form), and 1-methyldihydronicotinamide (MNAH, the reduced form) was used as comparison redox couple. MNAH can take part in A) acid-base and B) redox reactions. A) The dihydropyridine ring of MNAH can protonate, described by the following chemical equilibrium, protonation constant, and law of conservation of mass:


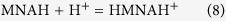



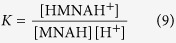



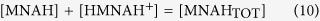


**Figure 6:**
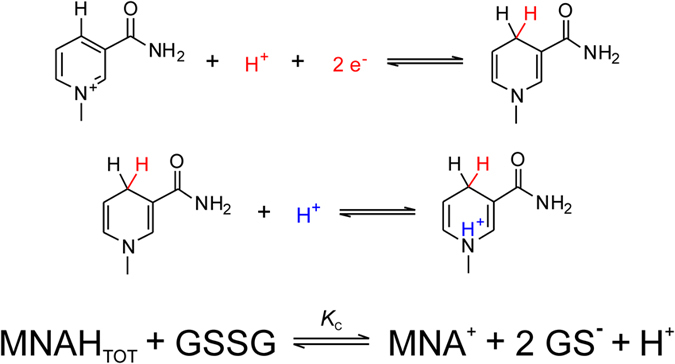
The redox and acid-base processes involving MNAH. The scheme of the redox equilibrium between MNAH and GSSG at the macroscopic level; here GS^-^ and GSSG represent the total sum of glutathione and glutathione disulfide species, respectively.

B) In redox reactions MNAH can be a two-electron reducing agent as exemplified in Fig. 6 with the disulfide form of glutathione.

The *K*_C_ equilibrium constant of the redox reaction is a pH-dependent, conditional (apparent) one:


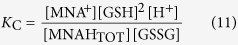


**Figure 7:**
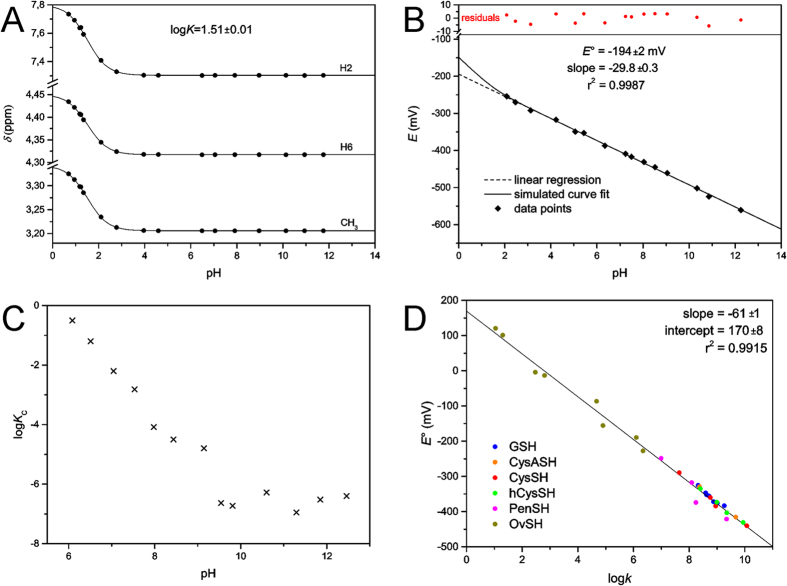
(**A**) The plot of ^1^H chemical shifts versus pH for MNAH; dots are data points, solid lines are simulated curve fits. (**B**) The pH dependence of the electrode potential (versus standard hydrogen electrode) of the MNA^+^/MNAH redox couple. (**C**) The pH dependence of the conditional redox equilibrium constant *K*_C_ (in log units) for the reaction between MNAH and GSSG. (**D**) The correlation between species-specific standard redox potentials and the species-specific thiolate protonation constants for the various thiols.

The oxidation and protonation of MNAH are interfering, codependent processes. We have therefore determined the protonation constant of MNAH by ^1^H NMR-pH titration (Fig. 7A). The resulting log*K* value (0.15 mol/L ionic strength, 25 °C) is 1.51 ± 0.01, which indicates that MNAH exists in solution at pH > 2.5 overwhelmingly in its neutral form.

The electrode potential of the MNA^+^/MNAH system was measured in a wide pH range against saturated calomel electrode, and then converted to redox potential values versus standard hydrogen electrode. In these measurements the MNA^+^ and MNAH_TOT_ concentrations were kept equal. The practical form of the Nernst equation (expressed with concentrations) for such a redox transition, where hydrogen ion also takes part in the reaction, is as follows:


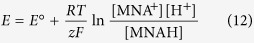


where *R* is the universal gas constant, *T* is the absolute temperature, *z* is the number of moles of electrons transferred in the half-reaction, and *F* is the Faraday constant. Based on the acid-base characterization of MANH, [MNAH] is practically equal to [MNAH_TOT_] in the pH media where the electrode potentials were measured, thus equation (12) takes the simpler form:


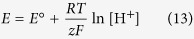


Therefore, the linear regression analysis of these data afforded the standard redox potential as the intercept; *E*°(MNA^+^/MNAH) = −194 ± 2 mV (Fig. 7B). Below this pH range however, the relative amount of [MNAH] decreases as it gradually protonates. In such an experimental setting, where [MNA^+^]/[MNAH_TOT_] is constant but the Nernstian term [MNA^+^]/[MNAH] is changing, the theoretical curve of the electrode potential versus pH is given by the following function, as [MNAH] can be expressed from equation (9):


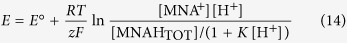


where *K* is the protonation constant of MNAH. Subsequently, since [MNA^+^] = [MNAH_TOT_] the above equation becomes:


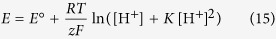


The curve of equation (15) is simulated on the redox potential data points (Fig. 7B) to demonstrate the deviation of theoretical curve from linear occurring only below pH 2. The linear regression analysis is therefore certainly valid in attaining the standard redox potential.

For the redox equilibrium between MNAH and GSSG, only the apparent or conditional equilibrium constants (*K*_C_) can be determined directly, by measuring the equilibrium concentrations of GSH, GSSG, MNA^+^, and MNAH_TOT_ in the reaction mixtures, using quantitative ^1^H NMR technique. These pH-dependent, conditional equilibrium parameters (compiled in Table 1, depicted in Fig. 7C) consist of total concentrations of the involved species, since the integrals of the observed NMR signals correspond to the total concentration of a reactant or product macrospecies.

The concentration of these macrospecies is actually the sum of the microspecies of the same number of bound protons, as shown in equation (7) for H_2_L^−^ of GSH. The pH-dependent, apparent constants can be decomposed into pH-independent, species-specific equilibrium constants. The number of the latter is certainly large, but definite. Determination of the *k* microscopic redox equilibrium constants from the *K*_C_ conditional equilibrium constants and related parameters is demonstrated below with the example of the *k*^B^ redox microconstant. Latter involves the B microspecies of reduced glutathione, and the corresponding E’ microspecies of glutathione disulfide and can be expressed in relation with the MNA^+^/MNAH system by the following equation:


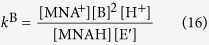


Note that the involvement of B necessitates the involvement of E′ (see Fig. 5), since these are the respective GSH and GSSG microspecies of identical side-chain protonation. The species-specific constants can be obtained as the product of total species concentration and the relative abundance of the respective microspecies. The relative abundance of the microspecies, in turn, is a function of pH and the microscopic protonation constants. For the B GSH microspecies, the concentration can be written as follows:


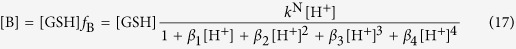


where [GSH] is the total solution concentration of glutathione, *f*_B_ is the relative abundance of microspecies B (i.e. *f*_B_ = [B]/[GSH]), *k*^N^ is the microscopic protonation constant of GSH involved in the formation of microspecies B, and *β* is the cumulative protonation constant of GSH. Substituting analogous equations as equation (17) for the concentrations of the microspecies in *k*^B^, one can write the equation of the microscopic equilibrium constant as follows:


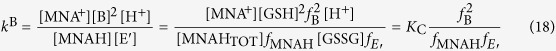


Thus, if *K*_C_, the apparent equilibrium constant and the *f* values are known the *k*^B^ value and all the analogous, pH-independent, species-specific redox microconstants can be calculated. The protonation macro- and microconstants of GSH and GSSG are taken from a previous work^21^ devoted to the species- and site-specific acid-base chemistry of glutathione. The species-specific standard redox potentials of the glutathione microspecies were calculated from the species-specific redox equilibrium constants using the Nernst equation, for example:


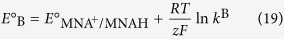


In principle, all of the conditional equilibrium constants measured at any pH media, would afford the calculation and the same value for a certain microscopic equilibrium constant. However, these calculations would be differently conditioned, therefore each microscopic redox equilibrium constant was evaluated from data at pH values where the corresponding equation (18) is well-conditioned, i.e. the mole fractions of the involved microspecies are near-maximal.

The species-specific standard redox potentials of the additional thiols were calculated using the species-specific redox equilibrium constants determined previously^14^,^18^, and the standard redox potential of glutathione microspecies B, since it bears the least uncertainty. The means of the calculated species-specific standard redox potentials are listed in Table 2. The correlation between the species-specific standard redox potentials and the species-specific thiolate protonation constants is depicted in Fig. 7D.

## Discussion

The highly interwoven acid-base and redox properties of the RSH-RSSR systems can be decomposed into elementary, component equilibria if a) the species-specific protonation constants are determined, and b) the conditional equilibrium constants of the pH-dependent redox equilibria are also quantified. In this work the standard redox potentials of six biogenic thiols are determined. As anticipated, the dissection of apparent equilibria into elementary redox ones in biological thiol-disulfide systems reveals significant differences between reactions of apparently highly similar, covalently identical reactants, improving thus our understanding of redox homeostasis and providing new means to influence it. The repertoire of species-specific standard redox potentials of glutathione improves our knowledge of the biochemistry of redox homeostasis and can lead to better interpretation of several biochemical phenomena. The example of GSH microspecies B and G (Table 2) shows that even minor changes in the sites of protonation can lead to differences in redox characteristics, this is all the more important in light of the complex, parallel protonation equilibria of these multibasic biomolecules: not only the protonation of the thiolate itself, its protonation fraction, and the concomitant redox behavior will be very sensitive to minor pH changes, but the protonation status of the adjacent basic moieties can influence the redox behavior by various inductive effects on the thiolate moiety. Concerning the systems studied, the GSSG/GSH redox potential shows modest variability, owing to the relative remoteness between the sulfur and the other acid-base functions in the molecule. The highest thiolate oxidizability of GSH (−383 mV) belongs to the microspecies in which all other basic sites in the molecule are in non-protonated form. The easiest reducibility of GSSG (−325 mV) is borne by the microspecies of complete amino and carboxylate protonation. By far the greatest versatility can be observed on ovothiol, encompassing as many as 348 mV differences between its species-specific limiting redox potential values. This astonishing variability is due to the fact that the adjacent basic sites are in the close vicinity of the thiolate. While the ovothiol versatility way exceeds even the differences between extrema of any arbitrarily selected different molecules, the actual ovothiol values indicate apparently low intrinsic reducing capability of OvSH. The fact, however, that OvSH exists as thiolate nearly throughout the pH scale, explains its wide pH range antioxidant potency compared to conventional thiols such as glutathione (latter is inherently a stronger reducing agent, but it exists mostly in the ‘inactive’ protonated form at neutral pH and below). The correlation between thiolate basicity and standard redox potentials verifies the previous claims that thiolate basicity and oxidizability are proportional parameters^4^,^11^.

The 30 standard redox potentials of the thiol-disulfide couples are determined for the first time; these values characterize the redox processes at the microspecies level. The 30 different species-specific equilibrium constant values provide now well-established means to predict thiolate oxidizabilities, a key parameter to understand and influence oxidative stress. The delicate task of introducing preventive or therapeutic antioxidants lies in their confinement; naturally small molecular reducing agents will never be as specific as the substrate-specific enzymes of the well-controlled antioxidant machinery of the cell, however with a finely tuned and designed thiolate basicity the redox potential and therefore the selectivity of the antioxidant thiol can be confined to a narrower range. The major difficulty in designing effective antioxidants without decomposing the physiological disulfides in biomolecules is that the redox potential of the latter could not so far be determined. The correlation between the redox and acid-base properties serves now as a sound basis to quantify reducibility of disulfide moieties in physiological proteins and peptides, allowing thus the development of potent, selective antioxidant compounds.

## Additional Information

**How to cite this article**: Mirzahosseini, A. and Noszál, B. Species-Specific Standard Redox Potential of Thiol-Disulfide Systems: A Key Parameter to Develop Agents against Oxidative Stress. *Sci. Rep*. **6**, 37596; doi: 10.1038/srep37596 (2016).

**Publisher’s note**: Springer Nature remains neutral with regard to jurisdictional claims in published maps and institutional affiliations.

## Figures and Tables

**Figure 1:**
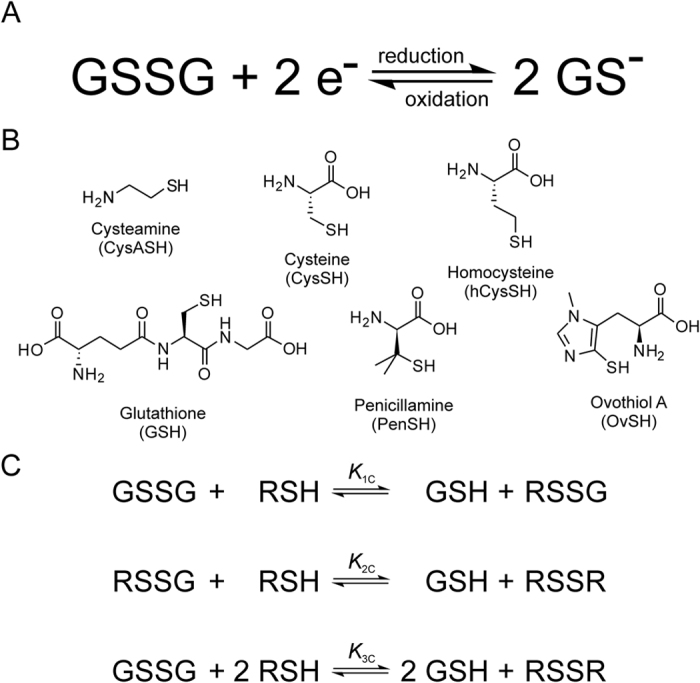
(**A**) The thiol-disulfide redox half-reaction between GSH and GSSG. (**B**) The structural formulae of the thiols studied. (**C**) Scheme of the thiol-disulfide equilibria at the macroscopic level. RSH denotes the thiols; RSSR denotes their respective homodisulfides; RSSG denotes their respective heterodisulfides formed with glutathione.

**Table 1:** Conditional redox equilibrium constants in log units of the reaction between MNA^+^ and GSH.

pH	log*K*_C_
6.08	−0.50
6.51	−1.21
7.04	−2.20
7.53	−2.82
7.98	−4.08
8.43	−4.50
9.14	−4.80
9.54	−6.64
9.81	−6.73
10.60	−6.28
11.30	−6.95
11.85	−6.52
12.46	−6.40

The uncertainties in the log*K*_C_ values are 0.02–0.04.

**Table 2:** Species-specific thiolate protonation constants and standard redox potentials of the corresponding thiol-disulfide systems.

microspecies		log*k*	*E*° (mV)	microspecies		log*k*	*E*° (mV)
GSH	A	9.26	−383	hCysSH	a	9.94	−431
	B	8.94	−374		b	8.99	−373
	D	8.87	−372		d	9.35	−403
	E	9.01	−376		f	8.4	−335
	G	8.59	−346	PenSH	a	9.34	−421
	H	8.71	−355		b	8.09	−317
	K	8.62	−352		d	8.24	−374
	N	8.32	−325		f	6.99	−248
CysASH	a	9.67	−416	OvSH	A	6.34	−227
	b	8.37	−329		B	2.8	−13
CysSH	a	10.07	−440		C	4.9	−155
	b	8.76	−360		D	6.1	−189
	d	8.95	−384		F	1.3	101
	f	7.64	−289		G	2.47	−4
					I	4.67	−87
					L	1.04	121

The one-letter symbols of microspecies are described in Figs 3, 4, 5. The uncertainties in the *E*° values are 5–10 mV.
